# Distinct association between aberrant methylation of Wnt inhibitors and genetic alterations in acute myeloid leukaemia

**DOI:** 10.1038/bjc.2011.471

**Published:** 2011-11-17

**Authors:** H-A Hou, Y-Y Kuo, C-Y Liu, M C Lee, J-L Tang, C-Y Chen, W-C Chou, C-F Huang, F-Y Lee, M-C Liu, M Yao, H-F Tien

**Affiliations:** 1 Division of Hematology, Department of Internal Medicine, National Taiwan University, Taipei, Taiwan; 2 Graduate Institute of Clinical Medicine, College of Medicine, National Taiwan University, Taipei, Taiwan; 3 Graduate Institute of Oncology, National Taiwan University, Taipei, Taiwan; 4 Biostatistics Consulting Laboratory, Department of Nursing, National Taipei College of Nursing, Taipei, Taiwan; 5 Department of Laboratory Medicine, National Taiwan University, Taipei, Taiwan; 6 Department of Pathology, National Taiwan University Hospital, National Taiwan University, Taipei, Taiwan

**Keywords:** Wnt pathway inhibitors, methylation, genetic alteration, acute myeloid leukaemia

## Abstract

**Background:**

Aberrant activation of Wnt signalling through hypermethylation of Wnt inhibitor genes is involved in several human malignancies, including acute myeloid leukaemia (AML). It remains unclear whether hypermethylation of Wnt inhibitors is associated with molecular gene mutations in the development of AML.

**Methods:**

We investigated the association of the promoter hypermethylation of six Wnt inhibitors (*Wif-1*, *SFRP1*, *SFRR2*, *SFRP4*, *SFRP5*, and *DKK1*) with gene aberrations in the leukaemogenesis of 269 AML patients.

**Results:**

In total, 166 patients (61.7%) had hypermethylation of at least one Wnt inhibitor. The majority (68.5%) of patients with Wnt inhibitor hypermethylation had concurrent Class II gene mutations that affect transcription factors or cofactors. There was a close association of *Wif-1* hypermethylation with t(15;17) (*P*=0.0005) and *CEBPA* mutation (*P*<0.0001), *DKK1* hypermethylation with t(8;21) (*P*<0.0001) and *ASXL1* mutation (*P*=0.0078), *SFRP-1* hypermethylation with t(8;21) (*P*<0.0001), *SFRP-2* hypermethylation with *AML1/RUNX1* mutation (*P*=0.0012), and *SFRP-5* hypermethylation with *MLL*/PTD (*P*=0.0505). On the other side, hypermethylation of Wnt inhibitors was always negatively associated with *NPM1* mutation and *FLT3*/ITD.

**Conclusion:**

There was distinct association between hypermethylation of individual Wnt inhibitors and specific gene aberrations, especially Class II mutations. The Wnt inhibitor hypermethylation might interact with genetic alterations in the leukaemogenesis.

Acute myeloid leukaemia (AML) is a heterogeneous group of haematological malignancies with great variability in the pathogenesis and clinical course. A two-hit model proposes that the development of AML requires cooperation between at least two classes of gene mutations; Class I mutations, such as *FLT3*, *RAS*, *JAK2*, *PTPN11*, and *KIT* mutations activate genes in the kinase signalling pathways conferring proliferation and/or survival advantage to haematopoietic cells and Class II mutations, such as *RUNX1/RUNX1T1*, *PML/RARα*, *CBFB/MYH11*, *MLL*/PTD, *AML1/RUNX1,* and *CEBPA* mutations, affect transcription factors impairing haematopoietic differentiation (Gilliland, 2002; McCormack *et al*, 2008; Renneville *et al*, 2008). Recently, inappropriate gene silencing through epigenetic modification, such as aberrant methylation in the promoter areas of suppressor genes, was also found to affect the development and progression of malignancies (Jones and Baylin, 2002). A link between genetic and epigenetic changes has been well demonstrated in colorectal cancer (Herman *et al*, 1998).

The Wnt pathway is highly conserved and instrumental in the embryogenesis and tissue maintenance (Reya *et al*, 2003; Willert *et al*, 2003). Activation of the Wnt/*β*-catenin pathway has been shown to be crucial for the establishment of normal and leukaemic stem cells (Austin *et al*, 1997; Reya *et al*, 2003; Wang *et al*, 2010). Dysregulation of Wnt signalling pathway is linked with several types of cancers, including AML (Korinek *et al*, 1997; Morin *et al*, 1997; Polakis, 2000; Chung *et al*, 2002; Clements *et al*, 2002; Reya and Clevers, 2005; Ysebaert *et al*, 2006). Chronic activation of Wnt pathway genes resulting from either aberrant overexpression of these genes or loss of natural Wnt inhibitors promotes uncontrolled cell growth and survival (Barker and Clevers, 2006). The Wnt signalling pathway is controlled by several natural Wnt inhibitors including Dickkopfs (DKK), secreted frizzled related proteins (SFRP), Wnt inhibitory factor-1 (Wif-1), human Dapper protein-1 (HDPR1), and so on. Abnormal activation of Wnt signalling by epigenetic silencing of these natural inhibitors was found in human cancers (Suzuki *et al*, 2004). Association between hypermethylation of some Wnt inhibitors and specific chromosomal translocations in AML was reported in a few studies (Chim *et al*, 2006; Suzuki *et al*, 2007; Jost *et al*, 2008), but not in others (Valencia *et al*, 2009). Furthermore, the association between hypermethylation of Wnt inhibitors and molecular gene alterations, especially the mutations recently detected, has not been reported. In this study, we aimed to investigate the role of hypermethylation of Wnt inhibitors in adult patients afflicted with AML. To the best of our knowledge, this is the first study to comprehensively address the association of hypermethylation of Wnt inhibitors with various genetic mutations in a large cohort of patients with *de novo* AML. We found distinct association between hypermethylation of certain Wnt inhibitors and specific genetic alterations, mostly Class II mutations, in the leukaemogenesis.

## Materials and methods

### Subjects

From April 1996 to June 2007, a total of 269 adult patients who were newly diagnosed as having *de novo* AML and had adequate samples for methylation analysis at the National Taiwan University Hospital (NTUH) were enrolled. Among 269 patients, 219 (81.4%) patients received intensive induction chemotherapy (Idarubicin 12 mg m^−2^ per day on days 1–3 and Cytarabine 100 mg m^−2^ per day on days 1–7) and then consolidation chemotherapy with 2–4 courses of high-dose Cytarabine (2000 mg m^−2^ q12 h days 1–4, total eight doses), with or without an anthracycline if complete remission (CR) was achieved (Tang *et al*, 2009; Hou *et al*, 2010). The remaining 50 patients received low-dose chemotherapy and/or supportive care because of the poor performance status or the patients’ will. This study was approved by the Institutional Review Board of the NTUH, and written informed consent was obtained from all the participants in accordance with the Declaration of Helsinki Principles.

### Methylation-specific polymerase chain reaction (MSP)

Bone marrow (BM) samples were obtained from all the patients at diagnosis. High-molecular-weight DNA was prepared from mononuclear cells as described previously (Tien *et al*, 1994). A CpG island is defined as a region with at least 200 bp and with a GC percentage greater than 50% and an observed/expected CpG ratio greater than 60% (Gardiner-Garden and Frommer, 1987). The primer sets for the analyses of Wnt inhibitor hypermethylation were designed by the help of the UCSC Genome Browser website (Kent *et al*, 2002), and the locations of these primers and the regions of the Wnt inhibitors analysed are shown in Supplementary Table 1. Methylation status of the CpG islands in the *SFRP1*, *SFRP2*, *SFRP4*, *SFRP5*, *HDPR1*, *Wif-1*, and *DKK1* gene promoters was determined by bisulfite treatment of genomic DNA followed by MSP as reported (Herman *et al*, 1996; Tien *et al*, 2001; Roman-Gomez *et al*, 2007). The method consists of two steps: modification of DNA using sodium bisulphite, which converts all unmethylated, but not methylated, cytosine to uracil, and amplification of the bisulphite-modified DNA by PCR using specific primers. Primer sequences of each gene for the unmethylated and methylated reactions are shown in Supplementary Table 2. Approximately 1 *μ*g DNA was sodium bisulphite-modified and subjected to MSP with primers specifically recognising the unmethylated or the methylated sequences of Wnt inhibitor, respectively, by EZ DNA Methylation-GOLD KIT (ZYMO Research, Orange, CA, USA). Polymerase chain reactions were run in a final volume of 25 *μ*l containing 20 ng bisulphite-treated DNA, 200 nmol l^−1^ deoxynucleotide triphosphate, 150 nmol l^−1^ of each primer, 1 U of AmpliTaq Gold polymerase and buffer (Applied Biosystems, Foster City, CA, USA). Polymerase chain reactions was carried out by heating at 95 °C for 10 min, followed by 35 cycles of 95 °C for 30 s, 61 °C for 30 s, and 72 °C for 30 s, with a final step for 10 min at 72 °C. Polymerase chain reaction products were separated on 2% agarose gels and visualised by Ethidium bromide staining. Raji cells treated *in vitro* with SssI methyltransferase (New England Biolabs, Beverly, MA, USA) in order to generate methylated DNA were served as a positive control and BM mononuclear cells from healthy BMT donors were used as negative controls.

### Cytogenetics

Bone marrow cells were harvested directly or after 1–3 days of unstimulated culture as described previously (Tien *et al*, 1995). Metaphase chromosomes were banded by trypsin-Giemsa technique and karyotyped according to the International System for Human Cytogenetic Nomenclature.

### Immunophenotype analysis

A panel of monoclonal antibodies to myeloid-associated antigens, including CD13, CD33, CD11b, CD15, CD14, and CD41a, as well as lymphoid-associated antigens, including CD2, CD5, CD7, CD19, CD10, and CD20, and lineage nonspecific antigens HLA-DR, CD34, and CD56 were used to characterise the phenotypes of the leukaemia cells as previously described (Tien *et al*, 1995; Chou *et al*, 2006).

### Mutation analysis

Mutation analyses of 13 relevant molecular marker genes, *NPM1* (Falini *et al*, 2005), *CEBPA* (Lin *et al*, 2005), *FLT3/ITD* (Shih *et al*, 2002), *FLT3/TKD* (Shih *et al*, 2004), *N-RAS*, *K-RAS* (Chen *et al*, 2006), *JAK2* (Chen *et al*, 2006), *KIT* (Chen *et al*, 2007), *AML1/RUNX1* (Tang *et al*, 2009), *MLL/PTD* (Shiah *et al*, 2002), *PTPN11* (Hou *et al*, 2008), *ASXL1* (Chou *et al*, 2010), and *WT1* (Hou *et al*, 2010) were performed as previously described.

### Statistical analysis

The association between chromosomal abnormalities/gene mutations and the Wnt inhibitor hypermethylation was analysed using the Monte Carlo simulation-based Fisher’s exact tests. That is, the statistical significance was calculated using a Monte Carlo simulation corrected for multiple hypothesis testing (each with 10 000 simulations and with a prior type-I error *α*=0.01). Mann–Whitney *U*-tests were used to compare continuous variables and medians of distributions. To evaluate the impact of Wnt hypermethylation on clinical outcome, only the 219 patients who received standard chemotherapy as mentioned above were included in the analysis. Overall survival (OS) was measured from the date of first diagnosis to the date of last follow-up or death from any cause, whereas relapse-free status indicated that the patient achieved CR and did not relapse by the end of this study. Kaplan–Meier estimation was used to plot survival curves, and log-rank tests were used to test the difference between groups. The Monte Carlo simulation-based Fisher’s exact tests were conducted using StatXact-8 (Cytel Inc., Cambridge, MA, USA), and other statistical analyses were performed with the SPSS 16 software (SPSS Inc., Chicago, IL, USA) and Statsdirect (Altrincham, Cheshire, UK).

## Results

### Methylation in promoters of Wnt inhibitors in AML patients

The frequencies of hypermethylation of Wnt inhibitors (in descending order) were as follows: 31.6% for *SFRP1*, 30.1% for *DKK1*, 26.0% for *Wif-1*, 19.3% for *SFRP2*, 12.6% for *SFRP5*, and 1.5% for *SFRP4*. Taken together, 166 AML patients (61.7%) had promoter hypermethylation of at least one *Wnt* inhibitor at diagnosis. A half (83 out of 166) of them had hypermethylation of two or more *Wnt* inhibitors. No abnormal methylation was found in *HDPR1*. Hypermethylation of at least one *SFRP* gene occurred in 40.1% (*n*=108) of the AML patients. Aberrant methylation of *SFRP1, SFRP2*, and *SFRP5* was closely associated with each other (all *P*<0.0001). *DKK1* hypermethylation frequently occurred concomitantly with hypermethylation of *SFRP* family (*P*<0.0001), but not *Wif-1* (*P*=0.7645).

### Clinical characteristics of patients with aberrant methylation of Wnt inhibitors

The comparison of clinical characteristics of patients with and without promoter hypermethylation of at least one Wnt inhibitor is shown in Table 1. Patients with FAB M0 subtype of AML had the highest incidence (100%) of hypermethylation of Wnt inhibitors, whereas those with M4/M5 subtype had the lowest incidence (47.3%, *P*=0.0006). For better delineation of the clinical impact of hypermethylation of specific Wnt inhibitor, we analysed the association of individual Wnt inhibitor hypermethylation with clinical characteristics, respectively (Table 2). *SFRP* hypermethylation occurred more frequently in the patients with AML M0 (87.5% of M0 patients *vs* 38.7% of others, *P*=0.0079), but was less in M4/M5 subtype (27.5% of M4/M5 *vs* 49.1% of others, *P*=0.0009). *DKK1* methylation was also more common in AML M0 subtype (75% *vs* 28.7%, *P*=0.0104). On the contrary, *Wif-1* methylation was preferentially found in AML M1 and M3 (42.1% of M1 *vs* 21.7% of others, *P*=0.0035 and 63.2% of M3 *vs* 23.2% of others, *P*=0.0005, respectively).

Male patients had a higher incidence of hypermethylation of Wnt inhibitors than females (62.0% *vs* 47.6%, *P*=0.023). Patients with hypermethylation of at least one Wnt inhibitor had lower WBC counts, blast counts and serum lactate dehydrogenase (LDH) levels at diagnosis. *SFRP-5* hypermethylation alone was associated with higher platelet counts (*P*=0.0144).

### Correlation between promoter hypermethylation of Wnt inhibitors and immunophenotypes of leukaemic cells

Promoter hypermethylation of any Wnt inhibitor as a whole was positively associated with the expression of HLA-DR (*P*=0.0385), CD19 (*P*=0.0026), and CD34 (*P*=0.005), but was inversely associated with the expression of CD14 (*P*=0.0237) on the leukaemic cells (Supplementary Table 3). There was no difference in the expression of other antigens between the patients with and without Wnt inhibitor methylation.

Acute myeloid leukaemia patients with hypermethylation of any *SFRP* gene as a whole had higher frequency of CD19 (*P*=0.0004), CD7 (*P*=0.0144), and CD34 expression (*P*=0.012), but had lower frequency of CD14 expression (*P*=0.0395) on the leukaemia cells. For individual SFRP, hypermethylation of *SFRP1* had similar pattern of association with antigen expression to that of *SFRP* as a whole (Supplementary Table 5). *SFRP2* hypermethylation showed no association with the expression of any antigen studied, whereas *SFRP5* hypermethylation had close association with CD11b and CD7 expression, but had inverse correlation with CD33 expression. On the other hand, *DKK1* methylation was positively associated with HLA-DR (*P*=0.0273), CD34 (*P*=0.0002), and CD56 expression (*P*=0.0238), and *Wif-1* methylation was positively associated with CD7 (*P*=0.0004), but was inversely associated with CD14 (*P*=0.0377) and CD33 expression (*P*=0.0383).

### Association of aberrant methylation of Wnt inhibitors with cytogenetic abnormalities

Chromosome data were available in 260 patients at diagnosis, including 157 with and 103 without hypermethylation of Wnt inhibitors (Table 3 and Supplementary Table 3). Hypermethylation of any Wnt inhibitor as a whole occurred more frequently in patients with favourable karyotype (79.4%) than in those with intermediate-risk (52.4%) or unfavourable cytogenetics (67.7%, *P*=0.0005), and more frequently in patients with abnormal cytogenetics than in those with normal karyotype (69% *vs* 50.8%, *P*=0.0034). It was also positively associated with t(8;21) (*P*=0.0014). Hypermethylation of any *SFRP* as a whole was closely associated with favourable cytogenetics (*P*=0.0132) and t(8;21) (*P*<0.0001), but negatively correlated with intermediate cytogenetics (*P*=0.0002) and normal karyotype (*P*=0.0072). For individual *SFRP*, hypermethylation of *SFRP1* had the same pattern of association with cytogenetic changes as that of *SFRP* as a whole (Table 3 and Supplementary Table 4). On the other side, hypermethylation of *SFRP2* was positively associated with unfavourable (*P*=0.0069) and complex cytogenetic (*P*=0.0006), but was inversely correlated with t(15;17) (*P*=0.0293). *DKK1* hypermethylation was detected more frequently in patients with favourable cytogenetics (*P*=0.0179) and t(8;21) (*P*<0.0001), but was less common in patients with intermediate cytogenetics (*P*=0.0002) and normal karyotype (*P*=0.0025). *Wif-1* hypermethylation was more frequently detected in the patients with t(15;17) than in other cytogenetic changes (63.2% of M3 *vs* 23.2% of others, *P*=0.0005), but was seldom found in the patients with t(8;21) (0% *vs* 29.7%, *P*<0.0001). There was no association of hypermethylation of Wnt inhibitors with other chromosomal abnormalities, including +8, +11, +13, +21, −5/del(5q), and −7/del(7q).

### Association of promoter hypermethylation of Wnt inhibitors with molecular gene mutations

Among the 166 patients with hypermethylation of any Wnt inhibitor, 145 (87.3%) patients showed concurrent molecular mutation of at least one gene at diagnosis (Table 4); 98 patients had one gene mutation, 35 had two, 10 had three and 2 patients had four gene mutations. In total, 100 patients (68.5%) had concurrently at least one Class II mutation, including *MLL*/PTD, *CEBPA*, and *AML1/RUNX1* mutations, t(8;21), t(15;17), inv (16), and t(11q23).

Patients with at least one Wnt inhibitor hypermethylation had a trend of higher incidence of *CEBPA* mutation than those without hypermethylation (16.9% *vs* 8.7%, *P*=0.0693), but had significantly lower incidence of *FLT3*/ITD and *NPM1* mutations (17.5% *vs* 34%, *P*=0.003 and 12.7% *vs* 36.9%, *P*<0.0001, respectively).

Among the 108 patients with *SFRP* methylation as a whole, 92 (85.2%) showed concurrent molecular gene mutations at diagnosis; 60 had one gene mutation, 23 had two, 7 had three, and 2 patients had four mutations. Sixty-nine (75.0%) of them had at least one Class II mutation concurrently. Patients with *SFRP* hypermethylation had a significantly lower incidence of *NPM1* mutation than those without the gene hypermethylation (11.1% *vs* 29.2%, *P*=0.0005, Table 4 and Supplementary Table 3). There was no difference in the incidence of other molecular gene mutations between patients with and without *SFRP* methylation. For individual *SFRP*, hypermethylation of *SFRP1* was negatively associated with *NPM1* mutation, whereas that of *SFRP2* was closely associated with *AML1/RUNX1* mutation, but negatively correlated with *NPM1* mutation, and *SFRP5* hypermethylation was positively associated with *MLL*/PTD (Table 4 and Supplementary Table 4).

Among the 70 patients with *Wif-1* methylation, 60 (85.7%) patients showed concomitant molecular gene mutations at diagnosis; 46 had one gene mutation, 10 had two and 4 patients had three. Of these, 38 (63.3%) had concurrently at least one Class II mutation. Patients with *Wif-1* hypermethylation had a significantly higher incidence of *CEBPA* mutation (28.6% *vs* 8.5%, *P*<0.0001, Table 4 and Supplementary Table 4), but had lower incidences of *FLT3*/ITD, *MLL*/PTD*, NPM1* mutations, and *ASXL1* mutations (14.3% *vs* 27.1%, *P*=0.0338; 0% *vs* 5.7%, *P*=0.0402; 12.9% *vs* 25.1%, *P*=0.043; and 2.9% *vs* 13.1%, *P*=0.0127, respectively) than those without the gene hypermethylation.

Among the 81 patients with *DKK1* hypermethylation, 70 (86.4%) showed concurrent gene mutations at diagnosis (Table 4 and Supplementary Table 4); 41 had one gene mutation, 23 had two, and 6 had three. Out of these, 52 (73.2%) had at least one Class II mutation concurrently. Patients with *DKK1* hypermethylation had a significantly higher incidence of *ASXL1* mutation (18.5% *vs* 6.9%, *P*=0.0078), but lower incidences of *FLT3*/ITD and *NPM1* mutations than those without the gene hypermethylation (14.8% *vs* 27.7% *P*=0.0284 and 9.9% *vs* 27.1%, *P*=0.0013, respectively).

### Impact of promoter hypermethylation of Wnt inhibitors on response to therapy and clinical outcome

Of the 219 AML patients undergoing conventional induction chemotherapy, 184 (84.0%) patients achieved a CR. With a median follow-up time of 32 months, the identified poorer prognostic factors for OS included older age (*P*<0.001), unfavourable cytogenetics (*P*<0.001), *FLT3/*ITD (*P*=0.03), and *AML1/RUNX1* mutations (*P*=0.039). However, hypermethylation of at least one Wnt inhibitor or methylation of any *SFRP* did not influence the CR rate, relapse rate, OS, and relapse-free survival (RFS). Further evaluation of the effect of hypermethylation of individual Wnt inhibitors, including *SFRP1, SFRP2, SFRP5, DKK1*, and *Wif-1* showed the same findings. Subgroup analyses in patients with favourable, intermediate, or unfavourable-risk cytogenetics, and in patients with normal karyotype or those with specific gene mutations could not demonstrate prognostic impact of hypermethylation of Wnt inhibitors. The number of genes hypermethylated also did not affect the treatment response.

## Discussion

In this study, we showed that aberrant promoter methylation of Wnt inhibitors was closely associated with specific cytogenetic abnormalities and molecular gene alterations in the patients afflicted with *de novo* AML. Most of the AML patients harbouring Wnt inhibitors hypermethylation had concurrent Class II mutations at diagnosis. Furthermore, aberrant Wnt inhibitor methylation was closely associated with male, FAB M0 subtype, lower WBC and blast counts and lower LDH levels, but inversely associated with M4/M5 subtype.

Few studies have focused on the correlation of hypermethylation of Wnt inhibitors with specific cytogenetic abnormalities. To the best of our knowledge, there has been no report concerning the association between hypermethylation of Wnt inhibitors and molecular gene mutations till now. This study recruited a large cohort of *de novo* adult AML patients for analyses of the association between abnormal promoter methylation of Wnt inhibitors and genetic alterations. We found that hypermethylation of *Wnt* inhibitors occurred predominantly in the patients with abnormal cytogenetics, especially in the favourable-risk group. In addition to the close association of *SFRP1* and *DKK1* hypermethylation with t(8;21) and *Wif-1* hypermethylation with t(15;17) (Tables 3 and 4), we found for the first time the close association of *Wif-1* hypermethylation with *CEBPA* mutation, *SFRP2* hypermethylation with *AML1/RUNX1* mutation, *SFRP5* hypermethylation with *MLL*/PTD, and *DKK1* hypermethylation with *ASXL1* mutation. These results further support the hypothesis that epigenetic alterations may cooperate with genetic alterations in the leukaemogenesis of AML. Muller-Tidow *et al* (2004) demonstrated AML-associated translocation products, such as RUNX1-RUNX1T1 and PML-RARα activated the plakoglobin production, resulting in the accumulation of endogenous β-catenin in the nucleus and further activation of relevant target genes. Results from this and other studies suggest that downregulation of Wnt inhibitors through promoter hypermethylation might be another mechanism leading to the activation of Wnt signalling pathway in AML with some chromosome translocations and gene mutations, such as t(8;21), t(15;17), *MLL*/PTD, *AML1/RUNX1*, *CEBPA*, and *ASXL1* mutations. The reason that these genetic alterations are closely associated with hypermethylation of specific Wnt inhibitors remains elusive.

Although we did not validate our findings by correlating the methylation status with the gene expression, downregulation of the *Wnt* inhibitors due to abnormal promoter methylation has been demonstrated previously (Roman-Gomez *et al*, 2007). Furthermore, methylation of Wnt inhibitors was shown to be associated with upregulation of the downstream signalling of the Wnt pathway (Roman-Gomez *et al*, 2007; Valencia *et al*, 2009). However, it remains unknown whether the hypermethylation of the Wnt inhibitors examined in this study indeed directly influence the Wnt canonical signalling pathway or the non-canonical pathway activities in AML because Bovolenta *et al* (2008) showed that *SFRP*s inhibit both Wnt canonical and non-canonical pathways and different *SFRP*s may have opposite effect on the same process. Re-expression of methylated Wnt inhibitors and inactivation of the Wnt pathway in the cell lines with aberrant methylation of these Wnt inhibitors were also shown after treatment with the demethylating agent decitabine (Valencia *et al*, 2009). These findings suggest that Wnt pathway is regulated, at least partially, by methylation of the Wnt inhibitors.

The percentage of patients with aberrant methylation of at least one Wnt inhibitor in this study (61.7%) was similar to that reported by Valencia *et al* (2009). However, the frequencies of aberrant methylation of *SFRP1*, *SFRP2*, *SFRP4*, and *SFRP5* (31.6%, 19.3%, 1.5%, and 12.6%, respectively; total 40.1%) in this study were lower than those (41%, 31%, 4%, and 22%, respectively) reported by Valencia *et al* (2009) who analysed 184 non-M3 AML patients, but were similar to those reported by Jost *et al* (2008). These probably reflect the difference in patient selection and ethnic diversity.

To investigate the prognostic relevance of abnormal methylation of Wnt inhibitors in AML patients, we focused on the patients receiving standard chemotherapy. However, we did not find the difference of clinical outcome, including CR rate, OS, and RFS, between AML patients with and without Wnt inhibitor hypermethylation. The same were also true in subgroups of patients with different risk cytogenetics or gene mutations. Jost *et al* (2008) also did not find any prognostic impact of aberrant methylation in *SFRP* promoters in 100 AML patients. Contrary to our findings, Chim *et al* (2006) pointed out that *Wif-1* methylation was an independent poor prognostic factor for DFS and Valencia *et al* (2009) showed AML patients with two or more methylated Wnt inhibitor genes had poorer RFS, but not OS, in the subgroup of patients 60 years or younger with intermediate-risk cytogenetics by multivariate analysis. Large-scale studies with more AML patients are needed to clarify this point.

In summary, our findings address that CpG island hypermethylation in the promoters of Wnt pathway inhibitors including *SFRP* family*, Wif-1*, and *DKK1* is a common event in AML. More intriguingly, there is distinct association between aberrant methylation of some Wnt inhibitors and specific genetic alterations, such as close association of *DKK1* hypermethylation with t(8;21) and *ASXL1* mutation, *Wif-1* hypermethylation with t(15;17) and *CEBPA* mutation, *SFRP1* hypermethylation with t(8;21), *SFRP2* hypermethylation with *AML1/RUNX1* mutation, and *SFRP5* methylation with *MLL*/PTD. Epigenetic alterations, such as hypermethylation of Wnt inhibitors, may interact with specific cytogenetic abnormalities or molecular gene mutations, especially Class II mutations, in the leukaemogenesis of AML.

## Figures and Tables

**Table 1:** Comparison of clinical and laboratory characteristics between AML patients with and without promoter hypermethylation of Wnt inhibitors

	***Wnt* inhbitors**		
**Characteristics**	**Methylated** a	**Non-methylated**	***P*-value**
Patient no.	166	103	
Age (years)^b^	47.5 (15–87)	48 (15–87)	0.7989
			
*Gender*			0.023
Male	103	49	
Female	63	54	
			
*FAB classification* c			0.0082
M0	8 (4.8)	0 (0)	0.0254
M1	39 (23.5)	18 (17.5)	0.2836
M2	54 (32.5)	31 (30.1)	0.6885
M3	15 (9.0)	4 (3.9)	0.1429
M4	37 (22.2)	41 (39.8)	0.0024
M5	6 (3.6)	7 (6.8)	0.2544
M6	4 (2.4)	2 (1.9)	>0.9999
M7	0	0	
Unclassified	3 (100)	0 (0)	
			
WBC ( × 10^6^ per l)^b^	15950 (310–352300)	54470 (300–627800)	*P*<0.0001
Blast ( × 10^6^ per l)^b^	7132 (0–348777)	32584 (0–456725)	*P*<0.0001
LDH (U l^−1^)^b^	859 (250–7734)	1273 (283–15000)	0.0003
Hb (g dl^−1^)^b^	7.9 (2.9–13.9)	7.8 (3.3–14)	0.6952
Plt ( × 10^9^ per l)^b^	40 (5–802)	46 (6–268)	0.2331

Abbreviations: AML=acute myeloid leukaemia; FAB=French-American-British; Hb=haemoglobin; LDH=lactate dehydrogenase; Plt=platelet; WBC=white blood cell.

a Hypermethylation of at least one *Wnt* inhibitor.

b Median (range).

c No of patients (%).

**Table 2:** Summary of correlation between hypermethylation of *Wnt* inhibitors and clinical and laboratory features

**Variables** a	**Wnt** b	**SFRP** c	**SFRP1**	**SFRP2**	**SFRP5**	**DKK1**	**Wif1**
Gender^d^	+(0.023)						
M0	+(0.0254)	+(0.0079)		+(0.0008)		+(0.0104)	
M1							+(0.0035)
M2			+(0.0114)				
M3				−(0.0298)			+(0.0005)
M4/M5	−(0.0006)	−(0.0009)	−(0.0083)				−(0.0019)
WBC^d^	−(<0.0001)	−(<0.0001)	−(<0.0001)	−(<0.0001)	−(0.0086)	−(0.0006)	−(0.0128)
Blast^d^	−(<0.0001)	−(0.0002)	−(0.0029)	−(0.0072)		−(0.0053)	−(0.045)
Plt^d^					+(0.0144)		
LDH^d^	−(0.0003)			−(0.0004)		−(0.0443)	−(0.0196)

Abbreviations: blank=no significant association; LDH=lactate dehydrogenase; plt=platelet; +=positive association (*P*-value); −=negative association (*P*-value).

a No significant correlation of hypermethylation of Wnt inhibitors with age and haemoglobin level, which were not shown in this table.

b Hypermethylation of any Wnt inhibitors including *SFRP*, *Wif-1*, and *DKK1*.

c Hypermethylation of any *SFRP* gene including *SFRP1*, *SFRP2*, *SFRP4*, and *SFRP5*.

d Gender (correlation with male); white blood cells, blast, platelet, and LDH (correlation with high level).

**Table 3:** Association of hypermethylation of *Wnt* inhibitors with chromosomal abnormalities^a^

	**No. of patients (%)**			**No. of patients (%)**			**No. of patients (%)**			**No of patients (%)**		
	**Wnt methylated** b	**Wnt unmethylated**		**SFRP methylated** c	**SFRP unmethylated**		**SFRP1 methylated**	**SFRP1 unmethylated**		**SFRP2 methylated**	**SFRP2 unmethylated**	
	***N*=158**	***N*=102**	***P*-value**	***N*=101**	***N*=159**	***P*-value**	***N*=82**	***N*=178**	***P*-value**	***N*=47**	***N*=213**	***P*-value**
Favourable^d^	50 (31.6)	13 (12.7)	0.001	34 (33.7)	29 (18.2)	0.0132	28 (34.1)	35 (19.7)	0.0196	7 (14.9)	56 (26.3)	0.0685
Intermediate^d^	87 (55.1)	79 (77.5)	<0.0001	52 (51.5)	114 (71.7)	0.0002	41 (50)	125 (70.2)	0.0029	28 (59.6)	138 (64.8)	0.2067
Unfavourable^d^	21 (13.3)	10 (9.8)	0.5575	15 (14.9)	16 (10.1)	0.3357	13 (15.9)	18 (10.1)	0.2187	12 (25.5)	19 (8.9)	0.0069
t(8;21)	27 (17.1)	4 (3.9)	0.0014	23 (22.8)	8 (5.0)	<0.0001	21 (25.6)	10 (5.6)	<0.0001	3 (6.4)	28 (13.1)	0.3175
t(15;17)	15 (9.5)	4 (3.9)	0.1414	6 (5.9)	13 (8.2)	0.6276	3 (3.7)	16 (9.0)	0.1979	0 (0)	19 (8.9)	0.0293
Inv(16)	8 (5.1)	5 (4.9)	>0.9999	5 (5.0)	8 (5.0)	>0.9999	4 (4.9)	9 (5.1)	>0.9999	4 (8.5)	9 (4.2)	0.2612
t(11q23)	4 (2.5)	6 (5.9)	0.1971	3 (3.0)	7 (4.4)	0.7449	1 (1.2)	9 (5.1)	0.1779	2 (4.3)	8 (3.8)	>0.9999
Normal	60 (38)	58 (56.9)	0.0034	35 (34.7)	83 (52.2)	0.0072	28 (34.1)	90 (50.6)	0.0158	17 (36.2)	101 (47.4)	0.1958
Complex	16 (10.1)	7 (6.9)	0.503	13 (12.9)	10 (6.3)	0.0765	11 (13.4)	12 (6.7)	0.0993	11 (23.4)	12 (5.6)	0.0006

	**No. of patients (%)**			**No. of patients (%)**			**No. of patients (%)**		
	**SFRP5 methylated**	**SFRP5 unmethylated**		**DKK1 methylated**	**DKK1 unmethylated**		**Wif-1 methylated**	**Wif-1 unmethylated**	
	***N*=31**	***N*=229**	***P*-value**	***N*=75**	***N*=185**	***P*-value**	***N*=68**	***N*=192**	***P*-value**
Favourable^d^	7 (22.6)	56 (24.5)	0.8294	27 (36)	36 (19.5)	0.0179	14 (20.6)	49 (25.5)	0.5128
Intermediate^d^	18 (58.1)	148 (64.6)	0.2644	36 (48)	130 (78.3)	0.0002	47 (69.1)	119 (62)	0.3181
Unfavourable^d^	6 (19.4)	25 (10.9)	0.2494	12 (16)	19 (70.3)	0.2995	7 (10.3)	24 (12.5)	0.828
t(8;21)	2 (6.5)	29 (12.7)	0.5523	20 (26.7)	11 (5.9)	<0.0001	0 (0)	31 (16.1)	<0.0001
t(15;17)	4 (12.9)	15 (6.6)	0.2584	2 (2.7)	17 (9.2)	0.1109	12 (17.6)	7 (3.6)	0.0005
Inv(16)	1 (3.2)	12 (5.2)	>0.9999	5 (6.7)	8 (4.3)	0.5302	2 (2.9)	11 (5.7)	0.5238
t(11q23)	0 (0)	10 (45.4)	0.6135	0 (0)	10 (5.4)	0.0673	2 (2.9)	8 (4.2)	>0.9999
Normal	14 (45.2)	104 (45.4)	>0.9999	23 (30.7)	95 (51.4)	0.0025	31 (45.6)	87 (45.3)	>0.9999
Complex	5 (16.1)	18 (7.9)	0.1679	9 (12)	14 (7.6)	0.3339	6 (8.8)	17 (8.9)	>0.9999

No significant correlation between hypermethylation of Wnt inhibitors and +8, +11, +13, +21, -7/7q^−^, and -5/5q^−^, which were not shown in this table.

The *P*-value was calculated using a Monte Carlo simulation corrected for multiple hypothesis testing (each with 10 000 simulations and with a prior type-I error *α*=0.01).

a Two hundred and sixty patients, including 158 *Wnt-*methylated and 102 *Wnt*-unmethylated patients, had chromosome data at diagnosis. Hypermethylation of SFRP4 was not included in the table because only 1.5% of patients had this change.

b Hypermethylation of any *Wnt* inhibitor including *SFRP*, *Wif-1*, and *DKK1.*

c Hypermethylation of any *SFRP* inhibitor including *SFRP1*, *SFRP2*, *SFRP4*, and *SFRP5.*

d Favourable, t(15;17), t(8;21), inv (16) ; unfavourable, -7, del(7q), -5, del(5q), 3q abnormality, complex abnormalities; Intermediate, normal karyotype and other abnormalities.

**Table 4:** Association of hypermethylation of *Wnt* inhibitors with molecular gene mutations^a^

	**No. of patients (%)**			**No. of patients (%)**			**No. of patients (%)**			**No of Patients (%)**		
	**Wnt**^b^ **methylated**	**Wnt unmethylated**		**SFRP**^c^ **methylated**	**SFRP unmethylated**		**SFRP1 methylated**	**SFRP1 unmethylated**		**SFRP2 methylated**	**SFRP2 unmethylated**	
	***N*=166**	***N*=103**	***P*-value**	***N*=108**	***N*=161**	***P*-value**	***N*=85**	***N*=184**	***P*-value**	**N=52**	**N=217**	***P*-value**
Any mutation^d^	145 (87.3)	87 (84.5)	0.5791	92 (85.2)	140 (87)	0.4673	74 (87.1)	158 (85.9)	>0.9999	40 (76.9)	192 (88.5)	0.0221
Class I	69 (41.6)	67 (65.0)	0.0003	42 (38.9)	94 (58.4)	0.0019	34 (40)	102 (55.4)	0.0255	20 (38.5)	116 (53.5)	0.0638
Class II	100 60.2)	37 (35.9)	0.0002	69 (63.9)	68 (42.2)	0.0005	55 (64.7)	82 (44.6)	0.0025	27 (51.9)	110 (50.7)	0.8788
FLT3/ITD	29 (17.5)	35 (34)	0.003	21 (19.4)	43 (26.7)	0.1904	14 (16.5)	50 (27.2)	0.0647	9 (17.3)	55 (25.3)	0.2776
CEBPA	28 (16.9)	9 (8.7)	0.0693	17 (15.7)	20 (12.4)	0.4729	15 (17.6)	22 (12)	0.253	5 (9.6)	32 (14.7)	0.5005
AML1/RUNX1	20 (12.0)	10 (9.7)	0.691	16 (14.8)	14 (8.7)	0.1657	10 (11.8)	20 (10.9)	0.8368	13 (25)	17 (7.8)	0.0012
MLL/PTD	6 (3.6)	6 (5.8)	0.5449	6 (5.6)	6 (3.7)	0.5522	5 (5.9)	7 (3.8)	0.5272	5 (9.6)	7 (3.2)	0.0596
NPM1	21 (12.7)	38 (36.9)	<0.0001	12 (11.1)	47 (29.2)	0.0005	8 (9.4)	51 (27.7)	0.0008	5 (9.6)	54 (24.9)	0.0155
ASXL1	17 (10.2)	11 (10.7)	>0.9999	8 (7.4)	20 (12.4)	0.4195	8 (9.4)	20 (10.9)	0.8315	6 (11.5)	22 (10.1)	0.8012

	**No. of patients (%)**			**No. of patients (%)**			**No. of patients (%)**		
	**SFRP5 methylated**	**SFRP5 unmethylated**		**DKK1 methylated**	**DKK1 unmethylated**		**Wif-1 methylated**	**Wif-1 unmethylated**	
	***N*=34**	***N*=235**	***P*-value**	***N*=81**	***N*=188**	***P*-value**	***N*=70**	***N*=199**	***P*-value**
Any mutation^d^	27 (79.4)	205 (87.2)	0.2825	70 (86.4)	162 (86.2)	>0.9999	60 (85.7)	172 (86.4)	0.6845
Class I	14 (41.1)	122 (51.9)	0.2738	33 (40.7)	103 (54.8)	0.0485	24 (34.3)	112 (56.3)	0.0021
Class II	20 (58.8)	117 (49.8)	0.3623	52 (64.2)	85 (45.2)	0.0052	38 (54.3)	99 (49.7)	0.5788
FLT3/ITD	9 (26.4)	55 (23.4)	0.6711	12 (14.8)	52 (27.7)	0.0284	10 (14.3)	54 (27.1)	0.0338
CEBPA	5 (14.7)	32 (13.6)	0.7939	12 (14.8)	25 (13.3)	0.8471	20 (28.6)	17 (8.5)	<0.0001
AML1/RUNX1	6 (17.6)	24 (10.2)	0.2386	12 (14.8)	18 (9.6)	0.2123	4 (5.7)	26 (13.1)	0.1218
MLL/PTD	4 (11.8)	8 (3.4)	0.0505	3 (3.7)	9 (4.8)	>0.9999	0 (0)	12 (6.0)	0.0402
NPM1	3 (8.8)	56 (23.8)	0.4827	8 (9.9)	51 (27.1)	0.0013	9 (12.9)	50 (25.1)	0.043
ASXL1	1 (2.9)	27 (11.5)	0.2238	15 (18.5)	13 (6.9)	0.0078	2 (2.9)	26 (13.1)	0.0127

Abbreviations: Class I, Class I mutations including *FLT3/*ITD, *FLT3/*TKD, *NRAS*, *KRAS*, *KIT*, *JAK2*, and *PTPN11* mutations; Class II, Class II mutations including *MLL/*PTD, *CEBPA*, and *AML1/RUNX1* mutations. t(8;21), t(15;17), inv (16), and t(11q23).

The *P*-value was calculated using a Monte Carlo simulation corrected for multiple hypothesis testing (each with 10 000 simulations and with a prior type-I error *α*=0.01).

a No significant correlation between hypermethylation of Wnt inhibitors and *FLT3/*TKD, *KIT*, *NRAS*, *KRAS*, *JAK2*, and *PTPN11* mutations, which were not shown in this table.

b Hypermethylation of any Wnt inhibitors including *SFRP*, *Wif-1*, and *DKK1*.

c Hypermethylation of any *SFRP* gene including *SFRP1*, *SFRP2*, *SFRP4*, and *SFRP5*.

d Any mutation included the mutation of any Class I, Class II, or others, such as *NPM1*, *WT1*, and *ASXL1* mutations.
