# Supplementary material for: Distinct association between aberrant methylation of Wnt inhibitors and genetic alterations in acute myeloid leukaemia
Source: Br J Cancer. 2011 Nov 17;105(12):1927–33. doi: 10.1038/bjc.2011.471 (PMC3251886; doi:10.1038/bjc.2011.471)
Supplement: Supplementary Tables 1–5 [file bjc2011471x1.doc]

**Supplementary Table 1**

**Extract genomic locations of the primer sets and the regions of Wnt inhibitors**

| Gene | Chromosome | Strand | Start | End |
| --- | --- | --- | --- | --- |
| WIF1-forward | 12 | - | 65515437 | 65515456 |
| WIF1-reverse | 12 | + | 65515305 | 65515327 |
| DKK1-forward | 10 | + | 54073983 | 54074003 |
| DKK1-reverse | 10 | - | 54074111 | 54074130 |
| SFRP1-forward | 8 | - | 41166955 | 41166974 |
| SFRP1-reverse | 8 | + | 41166860 | 41166880 |
| SFRP2-forward | 4 | - | 154710182 | 154710203 |
| SFRP2-reverse | 4 | + | 154710068 | 154710094 |
| SFRP4-forward | 7 | - | 37956501 | 37956520 |
| SFRP4-reverse | 7 | + | 37956293 | 37956313 |
| SFRP5-forward | 10 | - | 99531891 | 99531910 |
| SFRP5-reverse | 10 | + | 99531787 | 99531809 |
| HDRP1-forward | 14 | + | 59105121 | 59105141 |
| HDRP1-reverse | 14 | - | 59105272 | 59105299 |

**Supplementary Table 2**

**Primer Sequences of Wnt pathway inhibitors**

|  | Forward primer (5’-3’) | Reverse primer (5’-3’) |
| --- | --- | --- |
| DKK1 |  |  |
| M-MSP | TTAAGGGGTCGGAATGTTTC | CACGAAACCGTACCGATTC |
| U-MSP | TTTTAAGGGGTTGGAATGTTTT | CCACAAAACCATACCAATTCAAC |
| Wif-1 |  |  |
| M-MSP | CGTTTTATTGGGCGTATCGT | ACTAACGCGAACGAAATACGA |
| U-MSP | GGGTGTTTTATTGGGTGTATTGT | AAAAAAACTAACACAAACAAAATACAAAC |
| SFRP1 |  |  |
| M-MSP | GTTTTCGGAGTTAGTGTCGCGC | ACGATCGAAAACGACGCGAACG |
| U-MSP | GTAGTTTTTGGAGTTAGTGTTGTGT | ACCTACAATCAAAAACAACACAAACA |
| SFRP2 |  |  |
| M-MSP | TCGGAGTTTTTCGGAGTTGCGC | GCTCTCTTCGCTAAATACGACTCG |
| U-MSP | GGGTTGGAGTTTTTTGGAGTTGTGT | CCCACTCTCTTCACTAAATACAACTCA |
| SFRP4 |  |  |
| M-MSP | TCGAGGGGGAGTTCGCGT | CGCGAAATCCGACCGCGAA |
| U-MSP | GTTGAGGGGGAGTTTGTGT | AAACCACAAAATCCAACCACAAAAC |
| SFRP5 |  |  |
| M-MSP | TGGCGTTGGGCGGGACGTTC | AACCCGAACCTCGCCGTACG |
| U-MSP | TGGTGTTGGGTGGGATGTTTG | CAACCCAAACCTCACCATACAC |
| HDPR1 |  |  |
| M-MS | ACTACTAATCAAAAACGCCCTACG | AATAGTCGTGTTTTATTTTCGGGTAC |
| U-MSP | AAAACTACTAATCAAAAACACCCTACAC | ATAGTTGTGTTTTATTTTTGGGTATGA |

**Supplementary Table 3**

**Comparison of immunophenotyping between AML patients with and without hypermethylation of *Wnt* inhibitors***

| Antigen | Proportion (%) of patients with the antigen expression | | | P value |
| --- | --- | --- | --- | --- |
|  | Total patients | *Wnt*  methylated | *Wnt*  non-methylated |  |
| HLA-DR | 69.3 | 74.1 | 61.6 | 0.0385 |
| CD13 | 93.9 | 93.3 | 94.9 | 0.7911 |
| CD33 | 92.3 | 90.1 | 96.0 | 0.0976 |
| CD11b | 32.8 | 31.0 | 40.6 | 0.5941 |
| CD14 | 13.8 | 9.6 | 20.4 | 0.0237 |
| CD19 | 8.6 | 12.5 | 2.1 | 0.0026 |
| CD10 | 0 |  |  |  |
| CD7 | 18.5 | 21.9 | 13.1 | 0.0995 |
| CD2 | 2.3 | 3.1 | 1.0 | 0.4128 |
| CD15 | 47.3 | 47.5 | 46.9 | >0.9999 |
| CD34 | 64.9 | 71.4 | 54.1 | 0.005 |
| CD56 | 23.7 | 24.0 | 23.2 | >0.9999 |

*Hypermethylation of any Wnt inhibitor including *SFRP, Wif-1* and *DKK1*

**Supplementary Table 4**

**Summary of correlation between hypermethylation of Wnt inhibitors and genetic alterations, including cytogenetics and gene mutations**

| **Genetic change*** | **Hypermethylation** | | | | | | |
| --- | --- | --- | --- | --- | --- | --- | --- |
|  | **Wnt**^†^ | **SFRP**^‡^ | **SFRP1** | **SFRP2** | **SFRP5** | **DKK1** | **Wif1** |
| **Favorable** | ＋  (0.001) | ＋  (0.0126) | ＋  (0.0196) |  |  | ＋  (0.0065) |  |
| **Intermediate** | －  (<0.0001) | －  (0.0002) | －  (0.0029) |  |  | －  (0.001) |  |
| **Unfavorable** |  |  |  | ＋  (0.0069) |  |  |  |
| **Normal** | －  (0.0034) | －  (0.0072) | －  (0.0158) |  |  | －  (0.0025) |  |
| **t(8;21)** | ＋  (0.0014) | ＋  (<0.0001) | ＋  (<0.0001) |  |  | ＋  (<0.0001) | －  (<0.0001) |
| **t(15;17)** |  |  |  | －  (0.0293) |  |  | ＋  (0.0005) |
| **Cplx** |  |  |  | ＋  (0.0006) |  |  |  |
| **Class I** | －  (0.0003) | －  (0.0019) | －  (0.0255) |  |  | －  (0.0458) | －  (0.0021) |
| **Class II** | ＋  (0.0002) | ＋  (0.0005) | ＋  (0.0025) |  |  | ＋  (0.0052) |  |
| **FLT3/ITD** | －  (0.003) |  |  |  |  | －  (0.0284) | －  (0.0338) |
| **CEBPA** |  |  |  |  |  |  | ＋  (<0.0001) |
| **AML1/RUNX1** |  |  |  | ＋  (0.0012) |  |  |  |
| **MLL/PTD** |  |  |  |  | ＋  (0.0505) |  | －  (0.0402) |
| **NPM1** | －  (<0.0001) | －  (0.0005) | －  (0.0008) | －  (0.0155) |  | －  (0.0013) | －  (0.043) |
| **ASXL1** |  |  |  |  |  | ＋  (0.0078) | －  (0.0128) |

* No significant correlation between hypermethylation of *Wnt* inhibitors and +8, +11, +13, +21, -7/7q^-^, -5/5q^-^, inv(16), t(11q23), *FLT3/*TKD, *KIT, RAS, JAK2* and *PTPN11* mutations which were not shown in this table.

Favourable, t(15;17), t(8;21), inv (16) ; unfavorable, -7, del(7q), -5, del(5q), 3q abnormality, complex abnormalities; Intermediate, normal karyotype and other abnormalities

^†^ Hypermethylation of any *Wnt* inhibitor including *SFRP, Wif-1* and *DKK1*

^‡^ Hypermethylation of any *SFRP* inhibitor including *SFRP1, SFRP2, SFRP4* and *SFRP5*

Abbreviations: Class I, Class I mutations including *FLT3/*ITD*, FLT3/*TKD*, NRAS, KRAS, KIT, JAK2* and *PTPN11* mutations; Class II, Class II mutations including *MLL/*PTD*, CEBPA* and *AML1/RUNX1* mutations, t(8;21), t(15;17), inv (16) and t(11q23); ＋, positive association (P value); －, negative association (P value);

**Supplementary Table 5**

**Summary of correlation between hypermethylation of Wnt inhibitors and immunophenotype**

| **Antigen*** | **Hypermethylation** | | | | | | |
| --- | --- | --- | --- | --- | --- | --- | --- |
|  | **Wnt**^†^ | **SFRP**^‡^ | **DKK1** | **Wif1** | **SFRP1** | **SFRP2** | **SFRP5** |
| **HLA-DR** | ＋  (0.0385) |  | ＋  (0.0273) |  |  |  |  |
| **CD33** |  |  |  | －  (0.0383) |  |  | －  (0.0082) |
| **CD11b** |  |  |  |  |  |  | ＋  (0.014) |
| **CD14** | －  (0.0237) | －  (0.0395) |  | －  (0.0377) | －  (0.0504) |  |  |
| **CD19** | ＋  (0.0026) | ＋  (0.0004) |  |  | ＋  (0.0017) |  |  |
| **CD7** |  | ＋  (0.0144) |  | ＋  (0.0004) | ＋  (0.0251) |  | ＋  (0.029) |
| **CD34** | ＋  (0.005) | ＋  (0.012) | ＋  (0.0002) |  | ＋  (0.0076) |  |  |
| **CD56** |  |  | ＋  (0.0238) |  |  |  |  |

* No significant correlation between hypermethylation of Wnt inhibitors and CD2, CD10, CD13 and CD15 which were not shown in this table.

^†^Hypermethylation of any Wnt inhibitor including *SFRP, Wif-1* and *DKK1*

^‡^Hypermethylation of any *SFRP* gene including *SFRP1, SFRP2, SFRP4* and *SFRP5*

Abbreviations: HLA, human leukocyte antigen; CD, cluster designation;＋, positive association (P value); －, negative association (P value);
